# Long-term bio-power of ceramic microbial fuel cells in individual and stacked configurations

**DOI:** 10.1016/j.bioelechem.2020.107459

**Published:** 2020-06

**Authors:** Iwona Gajda, Oluwatosin Obata, Maria Jose Salar-Garcia, John Greenman, Ioannis A. Ieropoulos

**Affiliations:** a Bristol BioEnergy Centre, Bristol Robotics Laboratory, University of the West of England, BS16 1QY, UK; b Centre For Research in Biosciences, University of the West of England, BS16 1QY, UK

**Keywords:** Microbial fuel cell, Long-term operation, Stacking, Urine, Ceramic

## Abstract

In order to improve the potential of Microbial Fuel Cells (MFCs) as an applicable technology, the main challenge is to engineer practical systems for bioenergy production at larger scales and to test how the prototypes withstand the challenges occurring during the prolonged operation under constant feeding regime with real waste stream. This work presents the performance assessment of low-cost ceramic MFCs in the individual, stacked (modular) and modular cascade (3 modules) configurations during long term operation up to 19 months, utilising neat human urine as feedstock. During 1 year, the performance of the individual MFC units reached up to 1.56 mW (22.3 W/m^3^), exhibiting only 20% power loss on day 350 which was significantly smaller in comparison to conventional proton or cation exchange membranes. The stack module comprising 22 MFCs reached up to 21.4 mW (11.9 W/m^3^) showing power recovery to the initial output levels after 580 days, whereas the 3-module cascade reached up to 75 mW (13.9 W/m^3^) of power, showing 20% power loss on day 446. In terms of chemical oxygen demand (COD) removal, the 3-module cascade configuration achieved a cumulative reduction of >92%, which is higher than that observed in the single module (56%).

## Highlights

• Long-term assessment of different urine-fed MFC set-ups for practical applications.
• Long-term stability up to 19 months due to catholyte generation.
• Resilience and power recovery after starvation cycle in the parallel configuration.
• COD removal up to 92% due to cascading in MFC stack.
• Ceramic as low cost and durable membrane material for scaled-up systems.

## Graphical abstract

**Figure:**
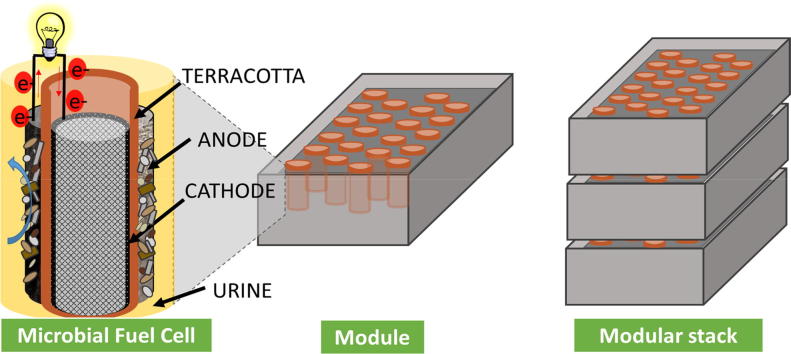

## 1 Introduction

Microbial Fuel Cells (MFCs) utilise organic feedstocks (wastewater, urine) as a fuel for direct electricity production by employing anode respiring microbes that convert organic matter into electrons while treating waste. In terms of effective applicability of MFC systems and the reduction of production costs, there is still much room for improvement in the reactor design and scalability process [23]. Many operational and design parameters, that might affect the power output by MFCs, can only be effectively tested in large-scale systems. Real-world implementation of MFCs requires that high power generation and treatment efficiency can be obtained with large-scale reactors, operated under realistic conditions [28], [30]. Different approaches can be employed for optimising MFC technology to allow their scale-up for practical applications and one being miniaturisation and multiplication of small-scale units [22] as it has been shown that higher energy density levels and optimum biofilm/electrode surface area–to–volume ratios reside within smaller scale MFCs. In order to scale-up MFC technology towards real-world applications and reach usable power levels, the MFCs units can be operated in collectives (stacks) of small-scale MFC units [19], [21], [33] using affordable and durable materials [7], [27]. Ceramic materials have shown to be suitable and cost-effective separators for MFCs [3], [41], [42]. Economic optimisation and the selection of the best stack structure becomes more essential at large-scale. It is also necessary to conduct sufficiently long-term experiments to determine and understand the long-term behaviour, stability and potential challenges. The choice of ceramic as a separator seems suitable for larger applications as over 60% of the material cost of the MFCs in the recent large scale application was due to the cation exchange membrane [16] therefore further study into the durability, performance and properties in long-term operation is much needed.

Additionally to the generation of electricity, MFC are used for breaking down and removing organic waste material from the processed substrate [17]. In this relationship between power and treatment, the higher the power output, the higher the rate of electron-abstraction from organic substrate that has been processed by the electroactive biofilm and the greater degree of waste removal [4], [45]. Over recent years more practical demonstrations have been reported [9]. However, little is known about their longevity under various operational conditions. The practical application of MFCs is currently restricted by the poor long-term stability of air cathodes, which has proven closely related to the scaling and biofilm growth on the cathodic catalyst layer [1], [2], [38]. As well as the cathode, also the separator suffers from blockages [10] due to precipitates causing transfer limitation of cations and decreased diffusion coefficients. However, this can be prevented by appropriate design and humidity control to allow for the catholyte production that is sustained by the electroosmotic drag washing the deposits away from the cathode surface [11], [14] and simultaneously producing antimicrobial agents [12]. In ceramic based pilot studies this approach showed potential for the remote power generation [5], [20] and it was implemented in small scale stack prototypes [15]. However, it is little known about the longevity of the system, its components and peripherals, and this study is aiming to address challenges ahead of the practical implementation and widespread distribution of the MFC technology in real-world scenarios in the future. The novel approach in this work uses low cost materials in MFC and performs a long-term evaluation which is rarely described in the literature. For this purpose, the long-term performance of the following MFC configurations: individual units, 22-MFC module and 3-module cascade in terms of power output and urine treatment capacity has been assessed. The tests were running for up to 19 months in fed-batch mode with real human urine and the stability of the systems and their resilience against adverse conditions were analysed.

## 2 Materials and methods

### 2.1 Individual MFC units configuration

**Fig. 1:**
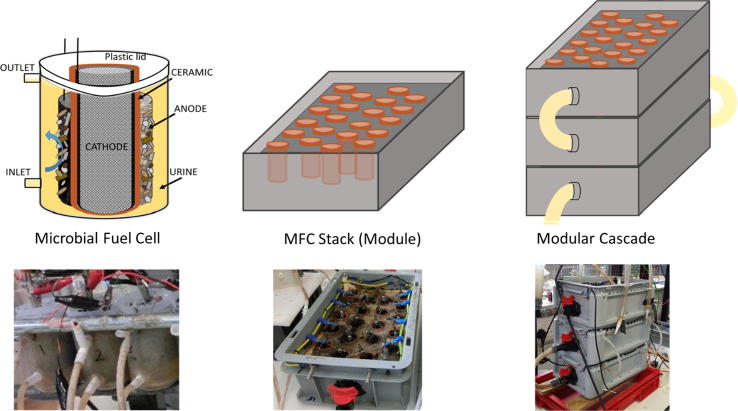
Experimental set up of the (i) individual MFCs (triplicate), (ii) assembled in 22-MFC module (stack) and 3-module cascade.

The individual MFC units used in this study were constructed from terracotta cylinders as described previously [13] using terracotta cylinders (H:50 mm) sealed at one end. The anode was made of 594 cm^2^ piece of carbon veil fibre (20 g/m^2^, PRF Composites, UK) which was folded and wrapped around the ceramic cylinder. Carbon veil is used here as a flexible substratum to support electrochemically active biofilm. The cathodes were prepared using activated carbon paste applied onto wet proofed carbon veil fibre as previously described [11]. The cathode with a total area of 22.5 cm^2^ was inserted into the inner chamber of the cylinder and connected with stainless steel crocodile clip. The MFC was hosted into the plastic lid that was holding the cylinder in place avoiding anolyte overflow (Fig. 1) and the lid was placed inside the polycarbonate plastic container. The anode chamber had plastic inlet connectors attached at the lower end of the plastic containers and T-connectors attached at the outlet to allow passive overflow of the anolyte. MFCs effective volume was 70 mL in each bioreactor. The anode and air-cathode were connected with stainless steel wire with an external resistance of 100 Ω. Continuous flow was maintained using a peristaltic pump (205U, Watson Marlow, UK) which was feeding urine from the inlet tank into the inflow tubes of the MFCs at a flowrate of 9 mL/h.

### 2.2 MFC modules

MFC-stack was assembled using Euro stacking container (Plastor, UK) of the dimensions 300 × 200 × 118 mm. The container was used as the chassis with attached pipes as the inlet and outlet to allow the electrolyte flow. 22-MFC units were installed inside the module using sealed acrylic lid holding each MFC in position with all the anode wires connected underneath to the main anodic connection on the outer side of the module. The MFCs were arranged in four rows where all the anodes and the cathodes were connected in parallel electrical configuration. The total volume of the anodic chamber was 1.8 L. All the cathodes were connected above the lid using stainless steel wires and two main connection cables on the sides of the module connected towards the main cathodic connection outside of the module (Fig. 1).

### 2.3 Modular cascade

Three modules as described above were stacked vertically using T-connectors and flexible tubing to allow the anolyte flow. The outlet of the first module was connected to the inlet of the second, whereas the outlet of the second module was connected to the inlet of the third module in the stack. The outlet of the third module was led into the outlet collection tank. Electrically, the stack was in parallel configuration using copper wires for the cathode parallel connection and stainless steel wire for the anodic connection. Main output from the 3-module stack was set up using a banana plug to alligator clip test lead cables connected to the decade box (ELC DR 05 Decade Box, RS, UK) and to the Agilent data acquisition equipment (Agilent LXI 34972A; Farnell, UK) and a PC to record voltage output.

### 2.4 Operation of MFC stacks and analysis

Anaerobic activated sludge, obtained from the Wessex Water wastewater treatment plant in Saltford, UK, was mixed 1:1 with urine and used as the inoculum. Urine was collected anonymously from healthy volunteers, pooled together and stored in a 40 L collection tank (pH ~ 9) at room temperature and used directly as the feedstock for the MFC modules in batch mode by daily replacement. For the chemical oxygen demand (COD) measurements, all samples were filtered through 0.45-μm syringe filters (Millex, US) to remove suspended solids from the media prior to further analysis. Polarisation curve experiments were performed using a potentiostat (SP-50, Biologic) by linear sweep voltammetry (LSV) from open circuit voltage (OCV) to 20 mV at a scan rate of 0.25 mV s^−1^. The MFCs were left in OCV for at least 2 h before performing the measurements to allow the stabilisation of the OCV. Polarisation curves were obtained by plotting the MFC voltage versus current (V vs. I) whereas power curves were obtained by plotting power versus MFC current (P vs. I) where the power was calculated by multiplying voltage and current.

## 3 Results and discussion

### 3.1 Long-term performance of the individual MFC-units

**Fig. 2:**
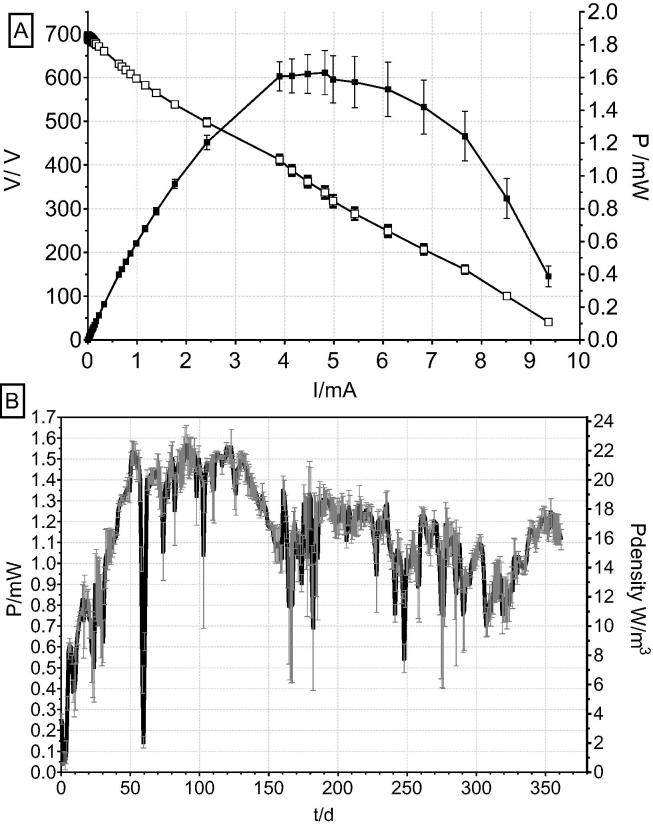
Polarisation (open) and power (solid) curves of the individual MFC units (A). Temporal performance of individual MFCs over 1 year of operation under continuous flow (B).

Polarisation of the systems were performed once the MFCs reached stability after 3 months of operation. The open circuit voltage (OCV) was 692 mV (Fig. 2A), reaching a maximum power output by the system of 1.63 mW at a current level of 4.81 mA. Regarding the temporal performance, the maximum value of power achieved was 1.56 mW corresponding to 22.3 W/m^3^ (Fig. 2B), which is similar value to previously reported data [13] in the same type and size of the MFC reactors. Individual MFC units were operated under continuous flow conditions and reached 1.55 mW of power on day 50, achieving stable output. On day 60, the power diminished due to feedstock depletion, after which the inlet tank was replenished with urine and the performance recovered to previous level, which is in agreement with previous work conducted in a continuous flow using human urine, reporting no power variance during 3 months of operating time [37]. As the feedstock flow and lab temperature were kept constant, the fluctuations must be related to the periodical feedstock depletion in the feeding tank as well as changes in feedstock composition, which is dependent on diet, time of the year and other factors. On day 350, the performance reached up to 1.24 mW, which is only 20% lower than the peak power output. These results suggest good reliability of the ceramic architecture in comparison to a commercial proton exchange membranes that reported 55% loss in performance in 6 months [34]. Membrane biofouling is an inevitable process in two-chambered MFCs utilising proton or cation exchange membranes [6] leading to physical blockage of charge transfer [43]. As it was shown, ceramic membranes offer robust and prolonged stability to perform to a similar level to CEM by the eighth month [41]. Moreover, in this study high performance, above 1 mW of power, is maintained even after 1 year of operation. Previous long-term studies have reported deteriorating performance levels also due to the cathode biofouling [1], [31] leading up to 55% power decrease in 1 year [44] as well as material degradation and change in bacterial microbiota [24].

In general, the power reduction over prolonged period of operation is much lower than in previously reported work [1], [10] as in the current study the long-term operation of MFCs was affected by the reduction of only 20% of power output after 350 days. This reduction in power might be caused by the increase in the thickness of the biofouling layer [8] on the membrane surface [6], [26], [34] as the accumulation of salt deposits were observed on the top edge of the ceramic cylinder that was exposed to air. However, the cathode chambers showed accumulation of liquid catholyte as previously described [12], [13], [14] that kept the inner cathodes sufficiently hydrated yet not completely flooded. The production of the catholyte maintained the cathode clean of any deposits in the long term operation of 1 year [14] and it might be due to antimicrobial properties [12] of the electrochemically produced liquid. It was also observed that the liquid kept the inner electrode clean of the salt deposits apart from the top part of the cylinder that was exposed to air. This might be the reason for good longevity of the system preventing from biofouling, scaling and clogging of the cathode.

### 3.2 Long term performance of the module

The performance of the MFC module in long-term operation was assessed during 19 months of fed-batch mode. The polarisation curve shows an OCV of 671 mV and the peak power at 21.5 mW (11.9 W/m^3^) achieved at a current level of 57.7 mA. The output is equivalent to the 0.98 mW per single MFC unit, however the total macro area of the anode in the individual test was double in size of the equivalent anode area per single MFC unit in the module. This is due to space limitations in the modular design restricting implementation of the 560 cm^2^ anodes.

**Fig. 3:**
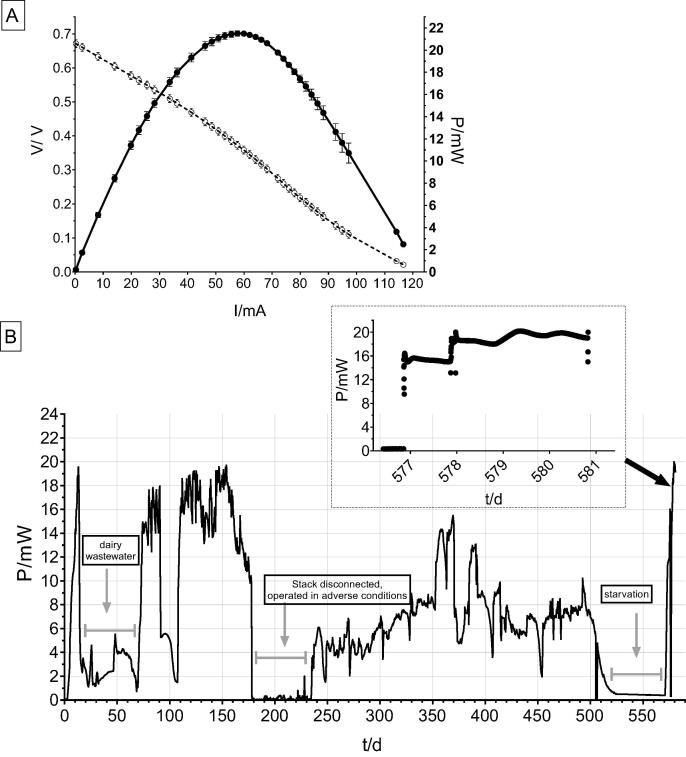
22-MFC stack arranged in Euro-Box Module. Polarisation (open) and power (solid) curves of the MFC module (A); long-term operation of 580 days under batch fed conditions with urine and dairy wastewater where indicated (B).

After the start of the experiment, the performance reached up to 19.0 mW when operated with urine. Afterwards dairy wastewater was tested (pH 5.5) for the period of two months and low power production was recorded in comparison to urine as a feedstock. When urine was reintroduced, the power output immediately increased and stabilised at the maximum level of approximately 19 mW after 100 days of operation. After 188 days of operating time the stack was removed from the data logger and connected to the 5 module cascade (data not shown) in the adverse conditions which might be the reason for the module underperformance that followed after day 235 when the stack was reconnected to the logger (Fig. 3). It showed some gradual recovery over the next 250 days, however the stack did not reach the initial power level. On day 395 the Constant Voltage Load (CVL) external circuitry was connected to the module where the voltage level was kept at 400 mV, dynamically adjusting the load as previously described [39], however this also did not improve the output. It might indicate the effect of the adverse conditions that changed the microbial diversity and the presence of non-electroactive microbes metabolising substrates to survive rather than generating current [35]. The stack was left for prolonged period of starvation after which it was refilled with fresh urine (Fig. 3, inset). The power level from the original stable performance was recovered. It might be due to the shift in bacterial community that was caused by the fuel deprivation. This fact might have changed the profile of the previously low performing microbial biofilm, formed probably as the outcome of the adverse conditions that changed towards mainly functioning as a shield rather than electroactive biofilm. This is similar to previously demonstrated resilience of the parallel configuration to prolonged starvation cycles and full recovery of the output [25]. Dynamic energy harvesting through the constant voltage adjustment kept throughout the starvation cycle might also contributed to re-shaping the electroactive microbiome on the anode [32]. The retention time in the individual MFCs was set to 7.5 h to allow achieving steady state of the system, however the operation of the module and the modular stack was in the batch-feeding mode where the retention time was usually 24 h (fed daily) up to 72 h (i.e. when not fed during the weekends) with visible periods of starvation. As the continuous operation requires a motorised pump for the feedstock supply, it is more suitable to implement the batch-mode feeding regime into the practical demonstrations and large scale set-ups.

Throughout the long-term study, it was observed that the cathodic wiring used for the parallel connections as well as stainless steel crocodile clips were corroded and needed to be replaced on multiple occasions. Corrosion was observed, however it was not affecting the current generation throughout the experiment. Corrosion and subsequent malfunction of the external wiring, as well as junctions, bolts and clips is a common issue in the long-term prototype testing [16] and it needs addressing in the development of future prototypes.

### 3.3 Long-term performance of the 3-Module cascade

**Fig. 4:**
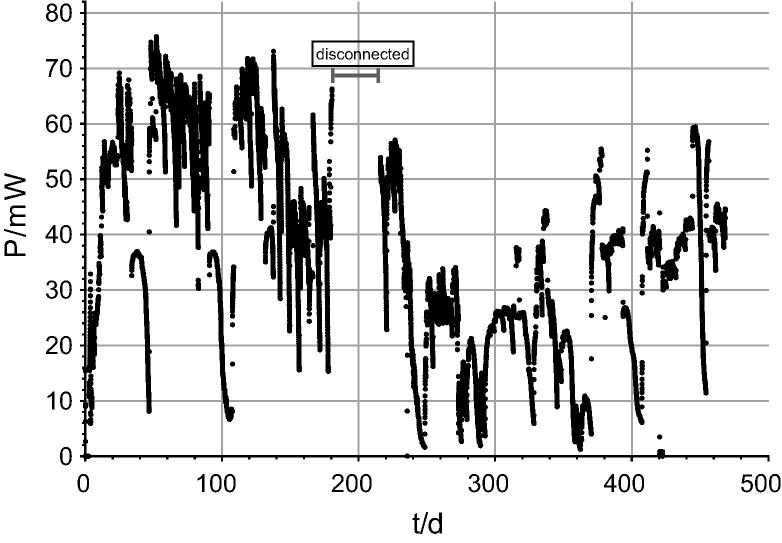
Long- term power performance of the 3-Module Cascade (66 MFCs in total) during 468 days of operation under batch feeding with urine.

The cascade was fed in batch mode and the external resistance was manually controlled starting with the 50 Ω, whereas 2 Ω was the optimum and 1.5 Ω was the heaviest load applied. Maximum performance reached 75 mW (13.9 W/m^3^) on day 52 since start (Fig. 4). Periodical occurrence of starvation periods shows the stack depletion in power and recovery when a new portion of feedstock was added. On day 446 (15 months of operation) it reached almost 60 mW of power which shows 20% lower output than the maximum (Fig. 4).

The efficient performance of the ceramic membrane confirms the suitability of the material [41] as a viable and inexpensive candidate for accelerating the scale-up process and wider use of the MFC technology. The generation of liquid catholyte keeps the moisture and humidity, sustaining ion transport for the cathodic electrochemical reaction without the accumulation of salt deposits on the cathode surface. System scale-up is being attempted as shown by the increasing number of studies including large-multi-panel cathodes [18], [36]. However, these suffer from severe inorganic fouling causing a more than 90% in power decline in the course of experiments [18].

### 3.4 Urine treatment capacity

**Fig. 5:**
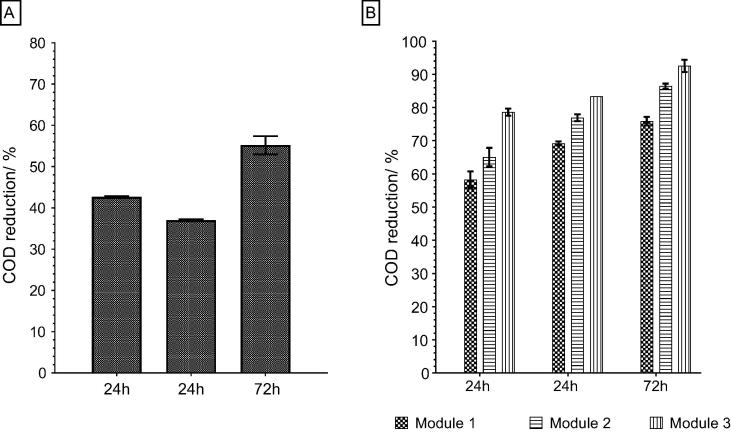
COD reduction in the module (A) and 3-Module cascade (B).

The treatment capacity of the module was assessed showing 42% of COD reduction in the 24 h and 37% during repeated test. Higher level of treatment was achieved during longer treatment period up to 55% of COD removal over 72 h (Fig. 5A). This treatment capacity was also tested in the modular cascade investigating stages of treatment between the modular components of the cascade. It was observed that during 24 h of operation the COD treatment reached 58%, 65% and 79% per Module 1, 2 and 3, respectively, and when repeated it shown 69%, 77% and 83% COD reduction. Over 72 h of treatment, the COD reduction increased from 76% in Module 1 to 86% in Module 2 and up to 93% in Module 3 (Fig. 5B). Part of the COD treatment would naturally be due to natural oxidation because the reported systems are aimed towards litre-scale practical applications where the effect of oxygen could not be avoided. The good levels of COD removal achieved in the modular system indicate that cascading is a good strategy for maximising the treatment capacity of these systems, in terms of COD removal [25] that can be adopted in the stacked configuration.

### 3.5 System characteristics and challenges

Table 1 provides the comparison between all three experimental set ups tested in this work and it is aiming to normalise the performance in metric and volumetric scale. The individual reactor is designed to have relatively small liquid volumes and thus a smaller electrode spacing due to large anode to cathode ratio (Table 1) however, this is not feasible for the development of the collective modules that are space limited. In this case, smaller anodes were used instead, lowering the anode to cathode ratio from 24.9 to 12.4. The performance of the stacked modules and their calculated volumetric power density shows improved output up to 13.9 W/m^3^, which is 5 times higher than the values obtained in similar configuration but using larger cylinders [20]. This result is one fold greater than the power reported in a previous work with similar size cylinder and similar module but different type of ceramic materials [15]. In general, the reduction of MFC size, variation in the type of ceramic as well as the improvement of the external circuitry resulted in an enhancement of the power density output by the system. The effect of the parallel connection on the microbial catalytic activity in MFC stacks suggests that it is a good strategy for long-term stability of stacked MFC systems.

**Table 1:** Characteristics of the individual MFCs, module and the modular cascade.

	Individual	Module	3-module Cascade
Anode area (cm^2^)	560	6160	18,480
Cathode (cm^2^)	22.5	495	1485
Anode to Cathode Ratio	24.9	12.4	12.4
Total Volume (mL)	70	1800	5400
Max. Raw Power (mW)	1.6	21.4	75.0
Max. Power density/Anode chamber (W/m^3^)	23.3	11.9	13.9
Max. Power density/Total Anode electrode (mW/m^2^)	29.1	34.7	40.6
Max. Power density/Projected Anode electrode (mW/m^2^)	417.9	249.4	291.4
Max. Power density/Cathode electrode (mW/m^2^)	724.4	432.3	505.1

The information in Schematic 1 shows the importance of peripherals in MFC scaled-up systems and the need for careful consideration of essential parts (such as resistive loads) to avoid significant detriment to the MFC performance. As can be seen in the example below, the effect of simply connecting the wires, which have to be corrosion-resistant and bio-compatible, can range from 1% to 33%, depending on scale and configuration, and this must be taken into account when designing appropriate circuitry for field applications.

**Schematic. 1:**
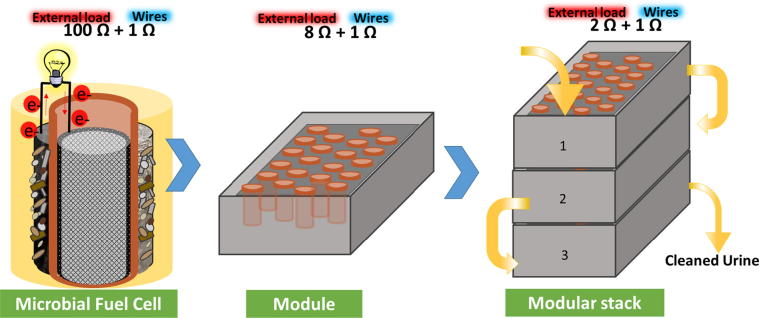
Electrical losses originating from the external wiring connected to the resistor load in all configurations.

Maximising power and improving longevity at decreased cost, is the key to promoting the MFC technology as a real product that can add value, through a range of practical applications [29], [40], [46] in a future with new markets.

## 4 Conclusions

Most of works in literature report MFC short-term assays, however for the purpose of practical application, it is crucial to test the performance of the system during prolonged operating times which would allow to address the potential challenges that might appear during the process. This work shows the long-term performance of three different MFC configurations utilising ceramic membranes and continuously fed with human urine. The behaviour of individual units during 1 year, showed power production up to 1.56 mW (22.3 W/m^3^), exhibiting significantly lower performance loss of only 20% in comparison to systems utilising conventional cation exchange membranes. A 22-unit stack produced up to 21.4 mW (11.9 W/m^3^) showing power recovery to the initial output levels after 580 days of operation, whereas the 3-module cascade (66 units in total) reached up to 75 mW (13.9 W/m^3^) of power, showing only 20% power loss. The results show that all MFC set-ups studied here are suitable for long-term processes, reporting lower loss of power compared with commercial membranes. In the case of the 3-modules cascade, the cascade configuration not only increased the power output but also the COD removal rate. Both good long-term stability, as well as the resilience of the system against changes in the operating conditions support the suitability of ceramic membranes for being used as a MFC separator, boosting the real-implementation of this technology.

## Declaration of Competing Interest

The authors declare that they have no known competing financial interests or personal relationships that could have appeared to influence the work reported in this paper.
